# A Novel Role for Histatin 5 in Combination with Zinc to Promote Commensalism in *C. albicans* Survivor Cells

**DOI:** 10.3390/pathogens10121609

**Published:** 2021-12-10

**Authors:** Hannah L. Norris, Rohitashw Kumar, Mira Edgerton

**Affiliations:** Department of Oral Biology, School of Dental Medicine, University at Buffalo, Buffalo, NY 14260, USA; hlnorris@buffalo.edu (H.L.N.); rohitash@buffalo.edu (R.K.)

**Keywords:** *Candida albicans*, Histatin 5, oral epithelia, innate immunity, stress-activated protein kinase signaling, cell wall, β-1,3 glucan, chitin, mannan, Interleukin-8, Interleukin-1β, Interleukin-10

## Abstract

*Candida albicans* is maintained as a commensal by immune mechanisms at the oral epithelia. Oral antifungal peptide Histatin 5 (Hst 5) may function in innate immunity, but the specific role Hst 5 plays in *C. albicans* commensalism is unclear. Since Zn-binding potentiates the candidacidal activity of Hst 5, we hypothesized that Hst 5+Zn would elicit a unique fungal stress response to shape interactions between *C. albicans* and oral epithelial cells (OECs). We found that Hst 5+Zn but not Hst 5 alone resulted in the activation of cell wall integrity (CWI) signaling, and deletion mutants were then used to determine that CWI-mediated chitin synthesis was protective against killing. Using flow cytometry, we confirmed that Hst 5+Zn-treated cells had significantly elevated levels of cell-wall chitin, mannan and β-1,3 glucan compared to Hst 5-treated cells. We then tested the activation of host signaling components involved in *C. albicans* cell-wall recognition. The immunoblot assay of *C. albicans*-exposed oral epithelial cells showed increased activation of EphA2 and NF-κB but not EGFR. Interestingly, *C. albicans* treated with Hst 5+Zn induced the global suppression of pro-inflammatory cytokine release from OECs, but an increase in negative regulator IL-10. Hst 5+Zn-treated cells were more adherent but ultimately less invasive to OECs than control cells, thus indicating lowered virulence. Therefore, Hst 5+Zn-treated *C. albicans* cells are discerned by epithelial monolayers, but are less virulent and promote anti-inflammatory signaling, suggesting that Hst 5+Zn in combination could play a role in regulating commensalism of oral *C. albicans* through cell wall reorganization.

## 1. Introduction

The salivary peptide Histatin 5 (Hst 5) is constitutively expressed in human saliva, has potent candidacidal activity for the opportunistic pathogen *Candida albicans*, and metal ion-binding ability including copper (Cu), iron (Fe), and zinc (Zn) ions [1,2,3]. These characteristics are supported by decades of research, yet the role of Hst 5 at the host-pathogen interface has remained understudied and poorly understood.

As an antifungal peptide, Hst 5 has long been postulated to function in the innate immune response to maintain a commensal microbiome. For example, Hst 5 is induced over 3-fold with early childhood caries [4], suggesting a role in responding to changes in the oral microenvironment. Furthermore, it has been suggested that Hst 5 is involved in host nutritional immunity [5] in the sequestration of metal ions away from invading pathogens due to its affinity for Zn (K_d_ = 1.2 × 10^−5^ M^−1^) [2]. However, the affinity of Hst 5 for Zn is orders of magnitude lower than other salivary Zn-binding proteins such as calprotectin (K_d_ = 1.4 × 10^−9^ M^−1^) [6], or the *C. albicans* zincophore Pra1 (K_d_ = 1.3 × 10^−9^ M^−1^) [7]. It is therefore unlikely that Hst 5 substantially contributes to fungicidal Zn sequestration at salivary pH. Despite this, Zn binding by Hst 5 has been shown by our group and others to be an interaction which could affect the in-situ function of Hst 5 by inducing intra-peptide compaction and dimerization of the peptide [8,9,10]. We also found that the addition of Zn at a ratio of 1 Zn to 2 Hst 5 potentiated the candidacidal activity of Hst 5, confirming that Zn induces a functional change in the peptide [10]. These zinc-binding characteristics bear similarity to zinc-regulatory proteins responsible for sensing and responding to intracellular zinc flux in human cells [11]. Since Hst 5 concentrations in saliva are approximately a 2:1 ratio with salivary zinc [12,13], it is clear that natural zinc flux from the external environment can have a profound effect on the mode of activity of Hst 5.

Hst 5 functions through an intracellular mechanism via uptake through the polyamine transporters Dur3 and Dur31 to induce ROS, cell cycle arrest, and non-lytic potassium and ATP efflux [14,15,16,17]. In contrast to this, we found that Zn increased the killing activity of Hst 5 through a pathway that results in rapid ATP efflux and does not require active uptake into fungal cells, consistent with fungicidal or fungistatic cell surface damage [18]. Zn increases the ability of Hst 5 to closely interact with membranes [19] while additionally promoting oligomerization [10], suggesting pore formation as a possible mechanism. Since physiological levels of Hst 5 in saliva do not kill all oral yeast, it is likely that these survivor *C. albicans* cells have undergone changes in their cell wall as a compensatory tactic following exposure with Hst 5. Therefore, we hypothesized that Hst 5+Zn would induce a different profile of mitogen activated protein kinase (MAPK) stress response than Hst 5, with an increase in the relative importance of cell-wall repair signaling. Though there are three stress-signaling MAPKs in *C. albicans* (HOG1, CEK1, MKC1), the response to cell-surface stress is primarily controlled by the cell wall integrity (CWI) pathway [20,21,22,23]. The CWI MAPK, Mkc1, is responsible for protective responses to a variety of cell-wall damaging agents, such as caspofungin, congo red, reactive oxygen species, and azoles [24]. The activation of Mkc1 is apparently dispensable for the immediate stress response to the membrane disrupting drug Amphotericin B (Amp B) [25], and yet high Mkc1 activation is seen in Amp B resistant cells [26] with the cell wall integrity pathway implicated in the fitness of Amp B-resistant mutants [27].

A major outcome of CWI signaling is cell wall chitin production through chitin synthase Chs3, which is under post-transcriptional control [25]. Furthermore, MAPK stress signaling induces changes in the geometry of cell-wall components β-1,3 glucan and mannan that can lead to altered host cell recognition of *C. albicans* cells [22,28,29]. Fungal β-1,3 glucan is generally considered to be the most important pro-inflammatory cell-wall component, and reduction in fungal glucan exposure can reduce host recognition [30]. Mannans are also recognized by host cells [28] and may also provide a barrier to limit access of fungicidal molecules to the plasma membrane [31]. The full role of chitin in recognition of *C. albicans* at the host epithelia is unclear and the subject of ongoing research [32], but it has been shown that purified *C. albicans* chitin upregulates production of the anti-inflammatory cytokine IL-10 in macrophages [33]. The rearrangement of the cell wall due to stress and other stimuli (i.e., treatment with a cell-surface damaging antifungal drug) can result in changes in the distribution of all 3 components at the cell surface and can cause unmasking of β-1,3 glucan [34]. Thus, changes in the abundance and expression of any cell wall component may impact other cell wall structures and alter host recognition.

Receptors and pathways involved in the recognition of cell-wall components vary by cell type. Surfaces such as the oral epithelia can be in continuous contact with commensal *Candida* and therefore have a different role in the recognition and response to fungal cell-wall components than immune cells [31]. Oral epithelial cells (OECs) are the first cell type in the oral environment that encounter *C. albicans* and are able to discern between the relatively benign yeast form and the more virulent hyphal form [35]. Consequently, receptors such as TLR2, TLR4, and Dectin-1 that are typically considered major *Candida* cell-wall component receptors in immune cells [22,36] do not elicit an inflammatory response in OECs but can be involved in general cell sensing and signal modulation [37]. In OECs, the receptor EphA2 is responsible for recognizing β-1,3 glucans in conjunction with heteromeric binding partner EGFR [38,39]. EGFR and EphA2 are mutually required for activation in response to *C. albicans* and lead to MAPK signaling that results in upregulation of cytokines, chemokines, and AMPs via the transcription factor c-Fos [35,39,40]. Though dectin-1 appears to play a reduced role in cell-wall sensing in OECs, signaling through NF-κB via dectin-1 does appear to contribute to the magnitude of cytokine release in response to β-glucans [38]. In any case, the overall signaling response of OECs to the fungal cell wall is highly variable due to the individual influence of the various polysaccharides.

Since Hst 5 is constitutively expressed in the oral environment, we theorized that Hst 5+Zn plays a role in maintaining a commensal relationship between *C. albicans* and host epithelial cells. To demonstrate the basis for such an interaction, we utilized an experimental model whereby *C. albicans* cells were pre-incubated with Hst 5 or Hst 5+Zn before seeding onto epithelial cell monolayers. A low-metal-binding, low-activity derivative of Hst 5, with ATCUN and HExxH motifs mutated to glutamine, herein named Hst 5ΔMB, was used as a comparison to understand the role of Zn binding on the downstream effects to the *C. albicans* cell wall. We found that Hst 5+Zn treated cells indeed had increased Mkc1 signaling and elevated cell surface expression of β-1,3 glucan, chitin and mannans compared to Hst 5 alone. Hst 5+Zn treated cells also elicited a distinct signaling response in oral epithelial cells that culminated in anti-inflammatory changes in cytokine release. Furthermore, these Hst 5+Zn treated cells had elevated host adhesion but were ultimately less invasive than cells treated with Hst 5 alone.

## 2. Results

### 2.1. Hst 5+Zn-Treated C. albicans Cells Have Increased Mkc1 Signaling

**Figure 1:**
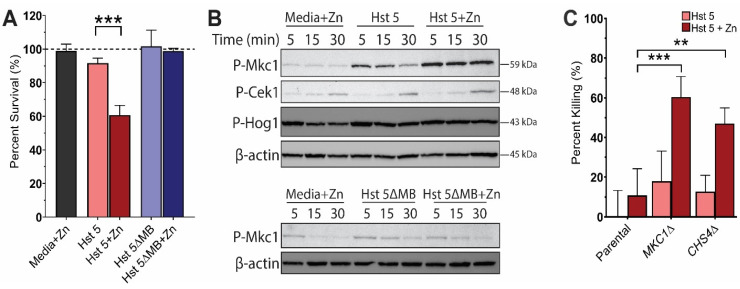
Hst 5+Zn treated *C. albicans* cells are protected by activation of the cell-wall integrity pathway. (**A**) Percentage survival of *C. albicans* cells treated with Hst 5 or Hst 5ΔMB (±Zn) in DMB media in 10 mM sodium phosphate buffer pH 7.4 for 1 h at 30 °C. Experiments were carried out on three separate days. (**B**) Immunoblot of MAPK activation time courses over 5, 15 and 30 min in *C. albicans* cells treated with DMB+Zn (Media+Zn), Hst 5, or Hst 5+Zn with blotting for P-Mkc1, P-Cek1, P-Hog1, or actin (Upper); or DMB+Zn (Media+Zn), Hst 5ΔMB, or Hst 5ΔMB+Zn blotting for P-Mkc1 and actin (Lower). (**C**) Percent killing *C. albicans* SN250 cells, *mkc1*/*mkc1* or *chs4/chs4* treated with Hst 5 (±Zn) in 10 mM sodium phosphate buffer pH 7.4 for 1 h at 30 °C. Experiments were carried out on 3 separate days. (** indicates *p* < 0.01, *** indicates *p* < 0.001, *n* = 3).

To determine the stress response of *C. albicans* cells that survive treatment with Hst 5 or Hst 5+Zn, we first assayed the survival of *C. albicans* after treatment with 3.15 fmol/cell Hst 5 or Hst 5ΔMB (± Zn) in dilute media buffer (DMB) (Figure 1A). At this dosage, we found that 92% of cells treated with Hst 5 survived compared to DMB-only control cells. As expected, the survival rate was lower in cells treated with Hst 5+Zn, but 61% still survived compared to control cells. Hst 5ΔMB and Hst 5ΔMB+Zn have almost no toxicity on *C. albicans* cells and had 99–100% survival under these conditions. We expected that Hst 5+Zn survivor cells would have a strong stress response that was distinct from cells surviving treatment with Hst 5 alone due to different mechanisms of action. We investigated the response of 3 major MAPK signaling pathways (Mkc1, Cek1 and Hog1) in cells treated with Hst 5 or Hst 5ΔMB with or without added Zn (Figure 1B). Hst 5-treated cells showed the strongest phosphorylation of Mkc1 at 5 min that subsequently decreased after 15–30 min, while yeast treated with Hst 5+Zn resulted in very strong and sustained P-Mkc1 from 5–30 min. Some increase in Cek1 phosphorylation occurred at 30 min in all conditions. No clear difference was observed between DMB+Zn control, Hst 5 or Hst 5+Zn with respect to Hog1 phosphorylation, although all conditions induced P-Hog1 compared to media alone (not shown). To determine whether P-Mkc1 was related to stress induced by antifungal effects of Hst 5, we examined cells treated with the low activity peptide Hst 5ΔMB or Hst 5ΔMB+Zn. Indeed, cells treated with this mutated peptide with or without added zinc did not elicit P-Mkc1 signaling.

As the MAPK of the cell wall integrity pathway, Mkc1 signaling is closely linked to protection from cell wall stress, including through upregulation of chitin production [23]. Therefore, we expected that knock-out of *MKC1* would result in *C. albicans* cells that are more susceptible to killing by Hst 5+Zn. We tested the candidacidal activity of Hst 5 and Hst 5+Zn in an Mkc1 homozygous deletion mutant (*mkc1*Δ/Δ) and a chitin synthesis mutant (*chs4*Δ/Δ) that is required for activation of an Mkc1-regulated chitin synthase (Figure 1C). A *C. albcans* *chs4* null mutant has been previously shown to have decreased chitin deposition compared to wild type cells [41]. When treating *mkc1*Δ/Δ cells with Hst 5 alone we found that there was not a significant change in killing compared with strain SN250. However, the combination of Hst 5+Zn significantly potentiated killing activity to 60%, suggesting that Mkc1 signaling is protective against Hst 5+Zn but dispensable for the response to Hst 5. Likewise, *chs4*Δ/Δ cells that lack Mkc1-dependent chitin synthase activity were not sensitive to Hst 5 at this dosage but were significantly more susceptible to killing by Hst 5+Zn, indicating that protection against killing afforded by Mkc1 signaling is mediated by chitin synthesis.

### 2.2. Hst 5+Zn-Treated Cells Have Higher Cell Wall Chitin, β-1,3 glucan, and Mannan

**Figure 2:**
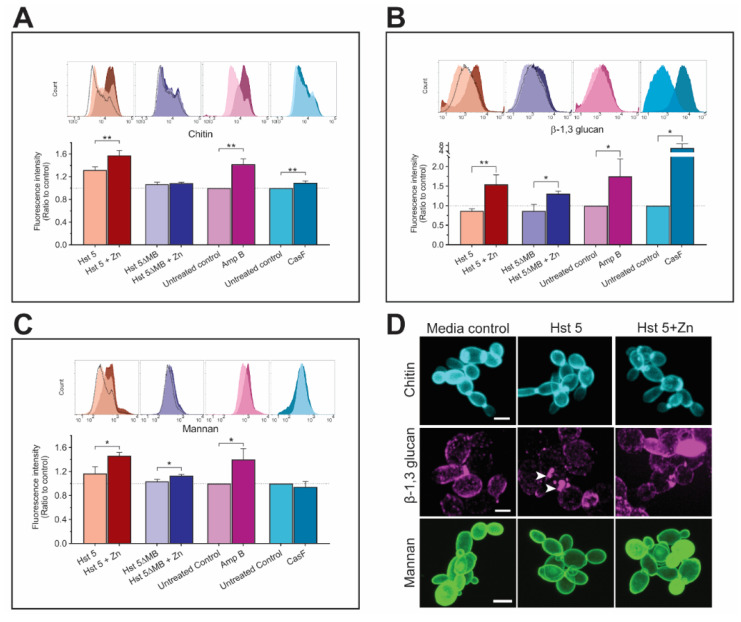
Hst 5+Zn survivor cells have higher cell wall chitin, β-1,3 glucan, and mannan. Fluorescently labeled cell wall components chitin (**A**), β-1,3 glucans (**B**) and mannans (**C**) were quantified via flow cytometry after 3 h of inducing conditions in DMB at 30 °C. Peak traces show a comparison of average fluorescence intensity between cells incubated in (left) DMB+Zn alone (dashed), or with Hst 5 (pink) and Hst 5+Zn (red); (left middle) DMB+Zn (dashed), or with Hst 5ΔMB (light blue), and Hst 5ΔMB+Zn (blue); (right middle) DMB alone (*Ca* control) (light purple) or with Amp B (purple); (right) DMB alone (*Ca* control) (light cyan) or with CasF (cyan). Peak shifts from left to right show increasing fluorescence intensity indicating an increase in the labeled cell wall component. Peak traces are representative data from one experiment. Bar graphs reflect fluorescence intensity averaged from three experiments and are expressed as a ratio with either DMB incubated control cells set to one, or DMB+Zn control cells in the case of Hst 5+Zn and Hst 5ΔMB+Zn. Experiments were carried out on three separate days. (* indicates *p* < 0.05, ** indicates *p* < 0.01, *n* = 3). (**D**) Confocal fluorescence images of stained chitin (top), β-1,3 glucans (middle) and mannans (bottom) after incubation for 3 h in DMB (left), Hst 5 (middle), or Hst 5+Zn (right). Scale bar = 4 microns.

As Mkc1 signaling results in cell-wall remodeling in response to stress, we anticipated that *C. albicans* cells that survived challenge with Hst 5 or Hst 5+Zn would exhibit changes in their cell wall structure. We assessed fluorescently labeled cell wall components using flow cytometry, specifically measuring chitin (Figure 2A), β-1,3 glucans (Figure 2B), and mannans (Figure 2C) following 3 h incubation with Hst 5, Hst 5+Zn and the low-activity mutant peptide Hst 5ΔMB. We additionally analyzed the cells treated with Caspofungin (CasF) as a positive control for induction of chitin synthesis and Amphotericin B (Amp B) to compare cell-wall changes under membrane-disrupting conditions.

Chitin staining was significantly (*p* = 0.0021) higher in Hst 5+Zn treated cells (1.6 fold to baseline) compared to Hst 5 (1.3-fold) although both treatments increased levels of chitin exposure (Figure 2A). However, chitin exposure was not altered following incubation with the zinc-binding mutant Hst 5ΔMB either with or without Zn. Chitin remodeling due to Hst 5 was similar to that resulting from Amp B treatment (1.4-fold, *p* = 0.0017) rather than cells treated with CasF (1.1-fold, *p* = 0.009), suggesting a high level of chitin exposure is induced in response to membrane disrupting drugs. The staining of β-1,3 glucan was unchanged in *C. albicans* cells treated with Hst 5 alone but was significantly (*p* = 0.0094) higher (1.5 fold to baseline) in Hst 5+Zn treated cells (Figure 2B). Cells treated with Hst 5ΔMB+Zn also had a small but significant (*p* = 0.0126) increase in staining (1.3-fold) compared with Hst 5ΔMB treated cells. Again, Hst 5+Zn treated cells showed most similarity to cells treated with Amp B (a 1.7-fold increase in β-1,3 glucan staining) compared to CasF-treated cells that had a 6× increase in β-1,3 glucan exposure. The overall profile of mannan exposure (Figure 2C) was similar to chitin staining in that Hst 5+Zn significantly (*p* = 0.0147) increased (1.5-fold) mannan exposure compared to Hst 5 (1.2-fold increase); while cells treated with Hst 5ΔMB+Zn had a small (1.1-fold) but significant (*p* = 0.0134) increase in stained mannan. Cells treated with Amp B had significantly (*p* = 0.0182) higher mannan than control (1.4-fold) but cells treated with CasF had no significant change in mannan staining. Overall, we found that Hst 5+Zn had a similar profile of cell wall changes as for Amp B, showing increased exposure in all 3 components measured. Hst 5+Zn treatment also resulted in significantly elevated exposure of these cell wall components compared to cells treated with Hst 5 alone.

Confocal fluorescence microscopy on *C. albicans* survivor cells treated with Hst 5 or Hst 5+Zn was carried out to visualize changes in surface localization compared to control cells (Figure 2D). The localization of chitin appeared to be similarly distributed between control cells and cells treated with Hst 5, with bright staining over the entire cell especially at the bud neck and scar regions. In cells treated with Hst 5+Zn, we observed a shift in chitin staining density that favored the bud neck and scars. In agreement with quantitation of β-1,3 glucan staining (Figure 2B), cells treated with Hst 5 appeared to have reduced overall levels of β-1,3 glucan although small areas of intense staining were visible (white arrows). Hst 5+Zn treatment resulted in many cells that had nearly uniform contiguous surface β-1,3 glucan exposure, as well as areas of intense patches such as those found in Hst 5 treated cells. We could not discern any qualitative differences in localization of mannan staining in control cells compared to those treated with Hst 5 or Hst 5+Zn. Thus, although cells treated with Hst 5+Zn quantitatively increased the exposure of all 3 cell-wall components to a similar degree, we observed the greatest change in surface morphology of β-1,3 glucan exposure compared to chitin or mannans.

### 2.3. Oral Epithelial Cells Incubated with Hst 5+Zn-Treated C. albicans Have Increased Cell Wall Signaling Response but Reduced Inflammatory Cytokine Release

**Figure 3:**
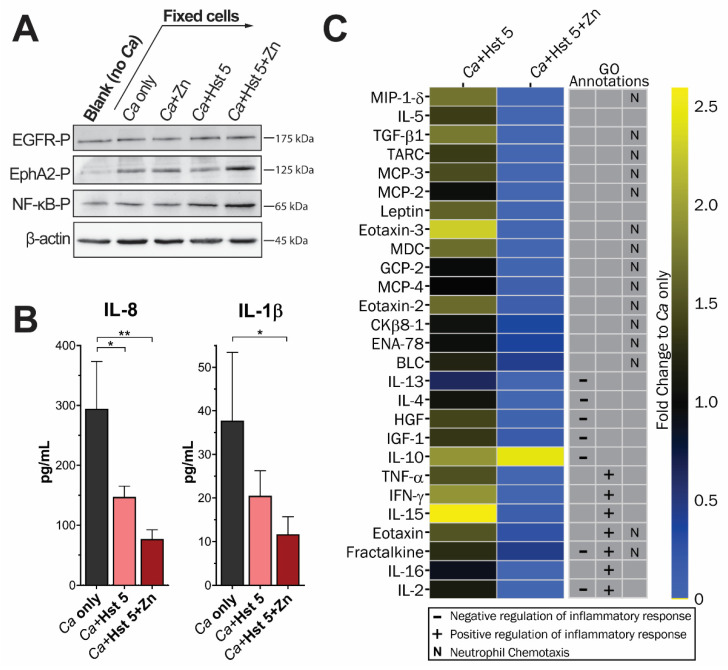
Oral epithelial cells incubated with fixed Hst 5+Zn treated cells have an altered epithelial signaling response. (**A**) Immunoblot of epithelial cell signaling activation after 1 h incubation with fixed *C. albicans* cells, with blotting for P-EphA2, P-EGFR, P-NF-κB, and β-actin. Monolayers were seeded with *C.albicans* cells incubated prior to fixation in: DMB alone (*Ca* only), 1.58 fmol/cell Zn, 3.15 fmol/cell Hst 5 (*Ca*+Hst 5), or 3.15 fmol/cell Hst 5 and 1.58 fmol/cell Zn (*Ca*+Hst 5+Zn). Experiment was carried out on 3 separate days. (**B**) ELISA of IL-8 (left) and IL-1β (right) release from TR146 monolayers after 24-h incubation with fixed yeast cells processed as above. Experiments were carried out on 3 separate days. (* indicates *p* < 0.05, ** indicates *p* < 0.01, *n* = 3) (**C**) Heat map of antibody array to detect cytokines released by TR146 monolayers after 24 h incubation with fixed yeast cells processed as above. Cytokine selection was performed in 2 parts: SLIM mapping was used to choose cytokines with GO annotations for innate immunity and inflammatory biological processes; then biologically relevant changes in OEC cytokine release due to Ca+Hst 5+Zn was set at a fold change in excess of ± 0.5 compared to control.

Since cell-wall reorganization can indicate changes in *C. albicans* virulence, we expected that the Hst 5+Zn survivor cell wall would induce signaling in oral epithelial cells and subsequent cytokine release. Monolayers of TR146 oral epithelial cells were seeded with methanol-fixed survivor cells for 1 h, then protein was collected and used for immunoblotting (Figure 3A). Protein was collected from *C. albicans*-naïve monolayers as a negative control. Phosphorylation of β-1,3 glucan receptor EphA2 and binding partner EGFR was assayed to indicate a response to β-1,3 glucan. Activation of NF-κB was also tested since this pathway is likely independent of EphA2 and is frequently implicated in signaling in response to the fungal cell wall. As expected, we found that Hst 5+Zn-treated *C. albicans* induced phosphorylation of EphA2 in epithelial cells compared to the other treatment conditions. However, EGFR phosphorylation in TR146 cells did not appear to be induced by Hst 5+Zn survivor cells. This suggests that Hst 5+Zn treated *C. albicans* mainly interacts with EphA2 but does not induce activation of EGFR. Phosphorylation of NF-κB also appeared to be elevated in oral epithelial cells (OECs) exposed to Hst 5+Zn-treated *C. albicans*, indicating that at least one other epithelial cell receptor interacts with the Hst 5+Zn-modified cell wall.

The activation of EphA2 is associated with the upregulation of IL-1β and IL-8 in OECs, therefore we measured their release from TR146 monolayers after 24 h incubation with pre-treated, fixed *C. albicans* (Figure 3B). For epithelial cells incubated with *C. albicans* control cells (gray bars), we found that IL-1β was released into the supernatant at a concentration of 37.8 pg/mL, while monolayers incubated with Hst 5 survivor cells released only 20.5 pg/mL. Epithelial cells incubated with Hst 5+Zn survivor cells released significantly (*p* = 0.0318) less IL-1β than the control at only 11.7 pg/mL. The cytokine IL-8 was tested in the same manner and was released into supernatant at 294.5 pg/mL by epithelial cells that were incubated with control *C. albicans.* Epithelial cells that were incubated with Hst 5-treated cells released significantly (*p* = 0.016) less IL-8 at 147.2 pg/mL, and monolayers incubated with Hst 5+Zn survivor cells released only 76.9 pg/mL, which was significantly (*p* = 0.0025) less than the control cells.

The significant decrease in IL-8 and IL-1β release was unexpected but suggested a net anti-inflammatory signaling response by OECs to Hst 5+Zn-treated *C. albicans*. We then utilized a cytokine array to probe a wider range of cytokines involved in inflammation and innate immunity, with a particular focus on positive and negative inflammatory regulation and innate immune cell recruitment (Figure 3C). Cytokines were selected as biologically relevant if levels changed ± 0.5-fold in *Ca*+Hst 5+Zn-incubated OECs compared to control. Interestingly, all inflammatory cytokines that exhibited a more than ± 0.5-fold change did so in a negative direction, excepting the potent negative regulator IL-10, which was increased 2.5-fold. Other than IL-10, the greatest changes in cytokine release in OECs incubated with *Ca*+Hst 5+Zn were seen in MIP-1-δ, IL-5, and TGF-β1, which decreased to undetectable levels compared to control OECs. As a counterpoint, epithelial cells incubated with Hst 5-treated *C. albicans* had similar or elevated cytokine release compared to OECs incubated with control *C. albicans* so that only 10 of the 27 selected cytokines exhibited a fold change greater than 0.5-fold and all were elevated compared to control. Elevated cytokines included MIP-1-δ, TGF-β1, Leptin, Eotaxin, Eotaxin-2, Eotaxin-3, MDC, IL-10, IFN-γ, and IL-15. These results suggest that epithelial cells differentiate between the cell surface of Hst 5- and Hst 5+Zn-treated *C. albicans* with distinct signaling that terminates in a unique profile of cytokine release. In the case of Hst 5+Zn-treated *C. albicans*, this signaling appears to be anti-inflammatory.

### 2.4. Hst 5+Zn-Treated Cells Are More Adherent but Less Invasive to Oral Epithelial Cell Monolayers

**Figure 4:**
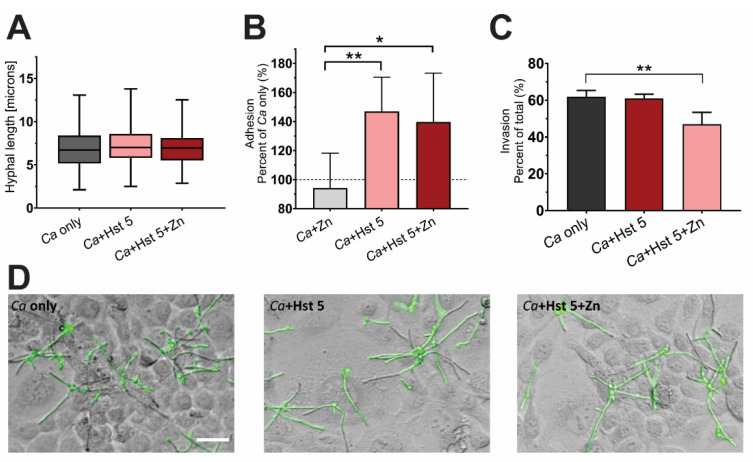
Hst 5+Zn treated cells are less invasive to oral epithelial cell monolayers and have altered adhesion. (**A**) Box and whisker blots showing hyphal length measurements after 30 min incubation of survivor cells on TR146 monolayers. Monolayers were seeded with DMB-incubated *C. albicans* (*Ca* only), or *C. albicans* pre-treated with Hst 5 or Hst 5+Zn. (*n* = 3) (**B**) Bar graph showing adhesion of fixed *C. albicans* cells as a percentage of media-incubated control cell (*Ca* only) adhesion to TR146 monolayers. *C. albicans* cells were incubated with DMB+Zn (*Ca+*Zn), Hst 5, or Hst 5+Zn. (* indicates *p* < 0.05, ** indicates *p* < 0.01, *n* = 6) (**C**) Bar graph showing quantitation of percent invaded *C. albicans* cells (*Ca* only), Hst 5 or Hst 5+Zn, as compared to total hyphae counts per field. Experiments were carried out on 2 separate days (** indicates *p* < 0.01, *n* = 4) (**D**) Representative merged fluorescence/brightfield images of *C. albicans* hyphal invasion on TR146 monolayers after 3 h of incubation at 37 °C. Cells were incubated with DMB (*Ca* only), or with added Hst 5 or Hst 5+Zn. Scale = 36 µm.

Since IL-10 has a major role in the host tolerance of commensals and pathogens, we questioned whether the upregulation of this cytokine indicated a push towards commensal interactions between yeast and host. In order to determine if this decrease in inflammatory signaling was indeed mutually beneficial, we tested *C. albicans* virulence markers including the ability of cells to form hyphae, adherence, and invasion of an epithelial monolayer (Figure 4). Since *C. albicans* hyphal production is essential for adhesion and invasion, we first assessed whether Hst 5-treated survivor cells have any impairment in hyphae formation (Figure 4A). Hst 5-treated *C. albicans* cells were seeded onto epithelial monolayers for 30 min, and hyphal growth was measured. We found no decrease in hyphal length between *Ca*-only control cells, or Hst 5 and Hst 5+Zn-treated cells (Figure 4A), suggesting that these cells were not impaired in the yeast-hyphae switch. Since changes in the cell wall can also lead to changes in the ability of *C. albicans* to adhere to surfaces, we tested the adhesion of fixed Hst 5-treated cells to epithelial monolayers after 1 h (Figure 4B). Both Hst 5 and Hst 5+Zn-treated cells had significantly (*p* = 0.0091, *p* = 0.023) increased adhesion to epithelial cells compared to *C. albicans* cells incubated in DMB+Zn, which adhered similarly to *Ca*-only control cells. Next, we measured whether increased adhesion corresponded with increased hyphal invasion of epithelial cells (Figure 4C,D). Control *C. albicans* cells incubated in DMB (*Ca* only) had invasion in 61.9% of total counts. Hyphae treated with Hst 5 had similar invasion (60.9% of total cells) while cells treated with Hst 5+Zn showed significantly decreased invasion (*p* < 0.01) at 47.0% of total cells. Thus, although Hst 5 and Hst 5+Zn-treated cells both had increased adherence to epithelial monolayers compared to control cells, only Hst 5+Zn treated cells had significantly reduced invasion ability.

## 3. Discussion

Fungal cells that survive exposure to antifungal drugs have altered host interactions, which are important to consider in the context of clinical treatment. A subpopulation of fungal cells can frequently survive antifungal drug treatment if the dosage or time of treatment is too low, and this can result in an enrichment of drug-adapted cells and acquired resistance over time [42]. In the case of endogenously produced Hst 5, the presence of commensal *Candida* in the oral environment confirms that levels in the saliva are low enough to allow a continued survival of stress-adapted *C. albicans* cells, even with enhanced activity due to Zn^2+^ binding. Therefore, it is important to understand whether these surviving cells have altered virulence and host interactions.

We found that Hst 5+Zn causes profound changes in stress signaling compared to Hst 5 alone, even at doses where a majority of cells survive. We also found that an increase in the activation of the CWI pathway is a unique protective response to Hst 5+Zn which functions via CWI-dependent chitin synthesis and leads to a marked increase in the level of, and changes in the distribution of cell-wall polysaccharides. The yeast cell wall is an important contributor to osmotic balance and is therefore a factor in recovery from membrane stress [43]. Since increased CWI signaling is a known adaptation to the membrane disrupting drug Amp B [26,27], we speculate that increased cell wall chitin improves the ability of *C. albicans* survivor cells to recover from membrane stress due to Hst 5+Zn. An increase in surface chitin, mannan and β-1,3 glucan was observed in both Amp B and Hst 5+Zn treated *C. albicans* cells, suggesting that these changes were a response to drug-induced membrane damage. Fluorescence microscopy further revealed large changes in the pattern of β-1,3 glucan exposure between cells treated with Hst 5 alone or Hst 5+Zn. A change in chitin distribution toward bud scars was also noted in cells treated with Hst 5+Zn, which is likely due to post-transcriptional upregulation of chitin synthase Chs3 via the CWI pathway. Both total exposure and the geometry of cell-wall components on the cell surface govern receptor interactions and host cell recognition [44], so we hypothesized that the changes observed in chitin and β-1,3 glucan could lead to altered recognition of cell-wall PAMPs (pathogen associated molecular patterns) and host signaling in response to contact with *C. albicans* cells.

Of the 3 cell-wall components considered in this study, β-1,3 glucan is the best understood in terms of interactions with the oral epithelia, as there is at least one known β-1,3 glucan receptor and 2 known β-1,3 glucan responsive signaling pathways [38,39], and a comparative paucity of information about the response to mannan or chitin. Therefore, we focused on aspects of oral epithelial cell (OEC) signaling which have evidence of responsiveness to fungal β-1,3 glucan: first, signaling through the receptor combination EphA2-EGFR [39]. Second, the transcription factor NF-κB which responds to various receptors but has sometimes been linked to Dectin-1 [38]. EphA2 was phosphorylated in response to Hst 5+Zn-treated cells, and NF-κB was also activated, suggesting that Hst 5+Zn-upregulated cell-wall moieties are recognized by EphA2 and at least one other receptor.

The activation of EphA2 by *C. albicans* in OECs has previously been linked to the production of IL-8, IL-1α, IL-1β, and CCL20 [38], but this requires concurrent signaling by EGFR which was not activated by exposure to *Ca*+Hst 5+Zn. We found that IL-8 and IL-1β release actually decreased in OECs exposed to *C. albicans* treated with Hst 5+Zn compared to control (*Ca* only) cells, which is consistent with the observed lack of EGFR activation. However, due to the parallel activation of NF-κB despite the outcome of lower cytokine release, we suspected that this was an inhibitory signaling event to negatively regulate inflammatory cytokine production. This interpretation was supported by the results of the larger cytokine array, which showed global decreases in pro-inflammatory cytokine release in conjunction with a distinct and unique increase in levels of IL-10. The cytokine IL-10 is a negative regulator of inflammatory cytokine production by blocking nuclear localization of NF-κB, though phosphorylation of the transcription factor is unaffected [45]. This clarifies how the apparent increase in NF-κB activation was not accompanied by canonical inflammatory cytokine production. It is unclear which signaling pathway leads to upregulation of IL-10 production in epithelial cells, however fungal chitin has been shown to mediate upregulation of IL-10 in peripheral blood mononuclear cells through concurrent activation of NOD2, TLR9, and mannose receptor [33].

The anti-inflammatory cytokine response to Hst 5+Zn-treated *C. albicans* led us to question whether these cells were more or less virulent. We assayed three aspects of fungal virulence using an OEC model. Hyphal length after 30 min was no different between *C. albicans* cells treated with DMB+Zn, Hst 5, or Hst 5+Zn, indicating that Hst 5+Zn survivor cells have no defect in the yeast-hyphae switch. Both Hst 5 and Hst 5+Zn survivor cells had increased adhesion to OEC monolayers compared to DMB+Zn treated controls, suggesting that these cells are less prone to dissemination in the oral environment. Since adhesion is closely linked to the hyphal switch and fungal recognition by OECs [46], increased adhesion combined with high exposed β-1,3 glucan likely contributes to the induction of EphA2. Finally, we found that Hst 5 and Hst 5+Zn survivor cells had decreased invasion of an OEC monolayer compared to DMB-incubated control cells, and Hst 5+Zn-treated cells were significantly less invasive than Hst 5-treated cells. Thus, despite an increase in OEC signaling in response to Hst 5+Zn-treated cells, these cells are less virulent than control cells.

We speculate that changes in cell surface chitin due to Hst 5+Zn treatment acts as a signal to OECs that *C. albicans* is growing less invasively, which results in decreased inflammatory cytokine release. This model is complimentary to a recent finding by Noble et al. (2021) that implicated the chitinase Cht2 as an important effector of commensalism in *C. albicans* [47]. Chitinases play a major role in cell-wall remodeling and process chitin molecules to different sizes [48], which modulates the effect on host cells [49]. The relationship between Cht2, chitin remodeling and host epithelial cell response is a topic which warrants further attention.

The ability to effectively treat candidiasis is dependent upon the relationship between the drug, the oral environment, and the stress response of *C. albicans*. Persistent, difficult to treat fungal infections are a serious concern that can elevate minor infections to life-threatening health issues. It is of great importance to fully understand the effect of fungicidal drugs on pathogenic yeast as a means of effectively maintaining a healthy homeostasis with the oral microbiota. Hst 5 in combination with Zn has potential as both a powerful physiological mechanism to maintain *C. albicans* in a commensal state as well as a tool for the effective treatment of oral candidiasis.

## 4. Materials and Methods

### 4.1. Yeast Strains, Media, and Culture Conditions

Experiments were carried out with *C. albicans* strain SC5314. Additional experiments were carried out with isogenic mutants *mkc1*/*mkc1* and *chs4/chs4* in parent strain SN152 (*his1Δ/his1Δ*, *leu2Δ/leu2Δ*, *arg4Δ/arg4Δ*, *URA3/ura3Δ::imm434*, *IRO1/iro1Δ::imm*), and reference strain SN250 (*his1Δ/his1Δ*, *leu2Δ::C.dubliniensis HIS1/leu2Δ::C.maltosa LEU2*, *arg4Δ/arg4Δ*, *URA3/ura3Δ::imm434*, *IRO1/iro1Δ::imm43*), derived from an SC5314 background [50]. Yeast-Peptone-Dextrose (YPD) media was used to culture yeast cells and YPD-Agar solid media was used for colony growth (Fisher Scientific, Waltham, MA, USA). All YPD media was prepared in deionized water with 50 µg/ mL supplemented uridine (Sigma-Aldrich, St. Louis, MO, USA). Briefly, *C. albicans* was cultured by inoculating a single colony into 20 mL YPD and grown 16 h at 30 °C with shaking at 220 rpm. Cultures were then diluted to OD_600_ of 0.3–0.4 and re-cultured to OD_600_ = 0.9–1.0. Cells were washed twice in 10 mM sodium phosphate buffer, pH 7.4 (NaPB) (Fisher Scientific, Waltham, MA, USA) prepared in HPLC water (JT Baker, Phillipsburg, NJ, USA). Cells were then diluted in NaPB to OD_600_ = 0.53–0.54 (1 × 10^6^ cells/mL) for candidacidal assay or OD_600_ = 0.99–1.0 (1 × 10^7^ cells/mL) for use in all other experiments. Metal salt solutions of ZnSO_4_·7H_2_O (Fisher Scientific, Waltham, MA, USA) were made in HPLC-grade water (JT Baker, Phillipsburg, NJ, USA). For MAPK activation and survivor cell experiments, dilute media buffer (DMB) consisting of Yeast Nitrogen Base media (0.17% YNB-ZnSO_4_ [Sunrise Science Products, Knoxville, TN, USA]/ 0.5% (NH_4_)SO_4_ [Fisher Scientific, Waltham, MA, USA]/ 2% Glucose [Sigma-Aldrich, St. Louis, MO, USA]) was prepared for use at a 25% dilution in 75% 10 mM NaPB.

### 4.2. Peptides

Histatin 5 (Hst 5) primary sequence (DSHAKRHHGYKRKFHEKHHSHRGY), and Histatin 5ΔMB (Hst 5 ΔMB) (QQQAKRHHGYKRKFQQQQQSHRGY that replaced both the N-terminal ATCUN motif (AA 1–3) and HExxH motif (AA 15–19) with glutamines) were synthesized by Genemed Synthesis, INC. (San Antonio, Texas, USA). Peptides were handled as previously described. A total of 36 mM Amphotericin B (Amp B) (Sigma-Aldrich, St. Louis, MO, USA) was prepared in 100% DMSO, and 3.6 mg/mL Caspofungin (CasF) (Sigma-Aldrich, St. Louis, MO, USA) was prepared in ddH_2_O.

### 4.3. Cell Survival Assays

Peptides (3.15 fmol/cell Hst 5 or Hst 5ΔMB) or an equal volume of NaPB buffer were mixed with or without 1.58 fmol/cell Zn^2+^, then incubated for 30 min at 25 °C. *C. albicans* SC5314 cells were added to samples at 1 × 10^7^ cells/mL in DMB. Cells and peptides were incubated at 30 °C with shaking at 220 rpm for 1 h. *C. albicans* cells were diluted in NaPB and plated on YPD-Agar. SN250 cells and deletion mutants were cultured as described above. Hst 5 (0.75 fmol/cell) or an equal volume of NaPB buffer was mixed with or without Zn^2+^ (37.5 fmol/cell), then incubated for 30 min at 25 °C. Aliquots of 2 × 10^5^ cells were added to samples and were incubated at 30 °C with shaking at 220 rpm for 1 h, diluted in NaPB, and plated on YPD-Agar. Cell survival was calculated by dividing colony counts of sample conditions by untreated controls. Percent killing was determined by taking the compliment of cell survival. Statistical differences between cell survival were calculated using ordinary one-way ANOVA with Sidak’s multiple comparison test.

### 4.4. C. albicans MAPK Phosphorylation

*C. albicans* SC5314 cells were cultured as described above. Peptides (0.79 fmol/cell) or an equal volume of NaPB buffer were pre-incubated for 30 min with or without 0.40 fmol/cell Zn^2+^. *C. albicans* SC5314 cells were added to pre-incubated samples at 1 × 10^7^ cells/mL in DMB buffer. Cells were incubated for 5, 15 or 30 min at 30 °C with shaking at 220 rpm before being harvested at 2500× *g*, then washed in 10 mM NaPB. Cell pellets were stored at −80 °C. Cells were lysed as previously described [20], and 7.2–7.5 µg protein was loaded on 12% SDS-PAGE gels and transferred onto PVDF (Biorad, Hercules, CA, USA). After transfer, membranes were blocked for 1 h at 25 °C in 5% bovine serum albumin (Sigma-Aldrich, St. Louis, MO, USA) in Tris-buffered saline with 0.1% Tween-20 (0.5 g BSA/10 mL TBS/T), followed by washing with TBS/T. Membranes were then incubated with primary antibodies at 4 °C overnight in 5% BSA TBS/T. Cek1 and Mkc1 phosphorylation were both detected using anti-phospho p42/44 MAPK ERK1/2 Thr202/Tyr204 rabbit polyclonal antibody as the primary antibody (Cell Signaling Technology, Boston, MA, USA). Hog1 phosphorylation was detected using an anti-phospho p38 MAPK Thr180/Tyr182 (D3F9) primary antibody (Cell Signaling Technology, Boston, MA, USA). Actin was detected using an anti-beta-actin polyclonal antibody (Bioss, Woburn, MA, USA). Membranes were then incubated with Goat anti-rabbit IgG-horseradish peroxidase (Cell Signaling Technology, Boston, MA, USA) secondary antibodies at 25 °C for 1 h in 5% BSA TBS/T. Chemiluminescent signals were generated using the SuperSignal West Pico detection kit (Thermo Scientific, Waltham, MA, USA) and detected using a ChemiDoc MP system (Biorad, Hercules, CA, USA).

### 4.5. Analyses of C. albicans Cell Wall Components

Antimicrobial agents were mixed in NaPB buffer (amphotericin B [0.05 fmol/cell]; caspofungin [0.26 fmol/cell]; Hst 5 and Hst 5ΔMB [3.15 fmol/cell], ± Zn^2+^ [1.58 fmol/cell]; or an equal volume of NaPB buffer) and incubated for 30 min at 25 °C. SC5314 cells were cultured as described above and added to samples at 1 × 10^7^ cells/mL in DMB buffer, then incubated for 3 h at 30 °C with shaking at 220 rpm. After incubation, cells were centrifuged at 2500× *g* at 4 °C and washed in NaPB. Samples were then fixed in 100% methanol for 20 min on ice (Fisher Scientific, Waltham, MA, USA). Fixed cells were centrifuged at 2500× *g* at 4 °C and washed in FCM staining buffer 0.5% BSA (Leinco Technologies, Inc., St. Louis, MO, USA). Staining of β-1,3 glucan was carried out with 1 mg/mL (1–3)-beta-glucan-directed monoclonal antibody (Biosupplies Australia Ltd., Bundoora, Australia) at 25 °C for 20 min with rocking before a 5 m incubation on ice. Cells were then washed 2 times at 2500× *g*, 4 °C. The secondary, anti-mouse IgG (H+L), F(ab’)2 Fragment (Alexa Fluor^®^ 647 Conjugate) (Cell Signaling Technology, Boston, MA, USA) was then added at a 1:50 dilution and cells were incubated on ice for 15 min. Samples were again washed 2 times at 2500× *g*, 4 °C and resuspended in FCM for flow cytometry. Mannan staining was carried out with 25 µg/mL Concanavalin A, Alexafluor 488 conjugate (ConA-488) (Life Technologies Corp., Carlsbad, CA, USA). Samples were incubated at 30 °C for 45 min with shaking at 220 RPM, then washed once at 2500× *g*, 4 °C, before being resuspended in FCM for flow cytometry. Chitin staining was completed using 25 µg/mL Calcofluor White (Sigma-Aldrich, St. Louis, MO, USA) Cells were allowed to sit at 25 °C for at least ½ h before flow cytometry was carried out.

Flow cytometry was carried out using the BD LSRFortessa flow cytometer. All treatment conditions were repeated on at least 3 separate days and average fluorescence intensity of at least 10,000 events was divided by the averages for same-day control cells incubated 3 h in either DMB or DMB+Zn. Baseline fluorescence intensity was set to 1. Statistical differences in average fluorescence intensity between control and treatment conditions (Hst 5 vs. Hst 5+Zn, Hst 5ΔMB vs Hst 5ΔMB+Zn, Media treated cells vs. Amp B or CasF) were calculated using unpaired *t*-tests. For fluorescent microscopy, *C. albicans* cells were stained as for flow cytometry and suspended in SlowFade™ Gold Antifade Mountant (Life Technologies Corp., Carlsbad, CA, USA). Samples were mounted on 25 × 75 mm microscope slides (Globe Scientific, Inc., Mahwah, NJ, USA) with 18 × 18 mm #1.5 cover glass (Fisher Scientific, Waltham, MA, USA) and sealed with nail polish. Images were acquired using an Andor Dragonfly spinning-disk confocal microscope (Oxford Instruments, Abingdon, UK).

### 4.6. Oral Epithelial Signaling in Response to the Fungal Cell Wall

SC5314 cells were cultured as above. Hst 5 (3.15 fmol/cell) or an equal volume of NaPB buffer, ± 1.58 fmol/cell Zn^2+^ was pre-mixed and incubated for 30 min at 25 ˚C. Cells were added to pre-mixed Hst 5 at 1 × 10^7^ cells/mL in DMB then incubated for 3 h at 30 ˚C with shaking at 220 rpm. then fixed in 100% methanol. TR146 buccal epithelial squamous cell carcinoma line was obtained from European Collection of Authenticated Cell Cultures (ECACC). TR146 cells have been characterized for use as a model for the human buccal epithelial barrier [38]. TR146 cells were routinely cultured in 1:1 DMEM/ F-12 medium supplemented with 10% FBS and maintained at 37 °C in a 5% CO_2_ humidified incubator. For standard experiments, TR146 epithelial cells were seeded at 1 × 10^5^ cells/mL in 12 well tissue culture plates or on acid washed 15 mm diameter glass coverslips previously placed in 12 well cell culture plates and cultured until the cells were confluent. Fixed *C. albicans* cells were seeded onto confluent epithelial cell monolayers at 1 × 10^7^ cells/well and incubated for 1 h at 37 °C. Oral epithelial cells (OECs) were lysed after treatment using 400 µL RIPA buffer (50 mM Tris-HCl, pH 7.4, 150 mM NaCl, 1 mM EDTA, 1% Triton X-100, 1% sodium deoxycholate, 0.1% SDS) containing complete protease inhibitor cocktail (Roche) and phosphatase inhibitors, left on ice for 30 min, and then centrifuged for 10 min at 21,000× *g* at 4 °C. Total protein concentration was determined using bicinchoninic acid (BCA) assay (Thermo Scientific, Waltham, MA, USA) and stored at −80 °C. For immunoblotting, total OEC lysate protein (20 µg) was separated by 12% SDS- PAGE and transferred to PVDF membrane (Biorad, Hercules, CA, USA). Membranes were blocked in 5% milk or BSA (Sigma-Aldrich, St. Louis, MO, USA) in Tris Buffered Saline pH = 7.2 containing 0.1% Tween-20 (TBST) at 20 °C for 1 h. Primary antibodies used were Phospho-NF-κB p65 (Ser536) (93H1) Rabbit mAb (1:1000), Phospho-EGF Receptor (Tyr1068) (D7A5) XP^®^ Rabbit mAb (1:1000), and c-Fos (9F6) Rabbit mAb (1:1000) (Cell Signaling Technology, Boston, MA, USA), as well as a β-Actin Polyclonal Antibody (1:1000) (Bioss, Woburn, MA, USA). Blots were incubated for 16 h at 4 °C. Membranes were washed twice with TBST and probed with Goat anti-rabbit IgG-horseradish peroxidase (Cell Signaling Technology, Boston, MA, USA) secondary antibody for 1 h at 25 °C. Secondary antibodies were detected using SuperSignal West Pico detection kit (Thermo Scientific, Waltham, MA, USA).

### 4.7. Cytokine Detection via ELISA and Antibody Array

SC5314 cells were cultured as above. Hst 5 (3.15 fmol/cell) or an equal volume of NaPB buffer, ± 1.58 fmol/cell Zn^2+^ was pre-mixed and incubated for 30 min at 25 °C. Cells were added to pre-mixed Hst 5 at 1 × 10^7^ cells/mL in DMB then incubated for 3 h at 30 °C with shaking at 220 rpm, then fixed in 100% methanol. Fixed *C. albicans* cells were seeded onto confluent epithelial cell monolayers at 1 × 10^7^ cells/well and incubated for 24 h at 37 °C. Supernatants from *Candida* treated oral epithelial cells were collected after 24 h and clarified at 21,000× *g* for 10 min to remove any cellular debris then frozen at −20 °C until the analysis was performed. Levels of secreted IL-1β or IL-8 in culture supernatants were determined using Human IL-1β and IL-8 ELISA MAX kits (Biolegend, San Diego, CA, USA) according to the manufacturer’s instructions. SoftMax Pro ELISA analysis software (Molecular Devices, San Jose, CA, USA) was used to calculate IL-1β or IL-8 concentrations using standard reference curves. Statistical differences between cytokine levels were calculated using ordinary one-way ANOVA with Dunnett’s multiple comparison test.

Culture supernatants were further applied to the RayBiotech Human Cytokine Antibody Array 5 according to the manufacturer’s instructions (RayBiotech, Inc. Norcross, GA, USA). SLIM mapping was carried out using the Generic GO Term Mapper (http://go.princeton.edu/cgi-bin/GOTermMapper, accessed on 4 November 2021) to choose cytokines with GO annotations for innate immunity and inflammatory biological processes [51]; then biologically relevant changes in OEC cytokine release due to *Ca*+Hst 5+Zn were set at a fold change in excess of ±0.5 compared to control.

### 4.8. Preparation of C. albicans Cells and TR146 Monolayers

SC5314 cells were cultured as above. Hst 5 (3.15 fmol/cell) or an equal volume of NaPB buffer, ± 1.58 fmol/cell Zn^2+^ was pre-mixed and incubated for 30 min at 25 °C. Cells were added to pre-mixed Hst 5 at 1 × 10^7^ cells/mL in DMB then incubated for 3 h at 30 °C with shaking at 220 rpm. Cells were either used immediately for invasion assays and hyphal growth assays or fixed in 100% methanol for use in adhesion assays. For all of the assays, TR146 oral epithelial cells were grown to confluence on 15 mm glass coverslips for 48 h in 12 well tissue culture plates, and were serum starved overnight prior to experiments before *C. albicans* cells were seeded at 1 × 10^5^ cells/well in 1 mL serum-free DMEM/F12 medium.

### 4.9. Invasion, Hyphal Growth and Adhesion

For the invasion assays, *C. albicans* seeded monolayers were prepared as described above and incubated for 3 h at 37 °C. After incubation, non-adherent *C. albicans* cells were removed and washed once with 1 × PBS and fixed with 4% formaldehyde. External portions of *C. albicans* hyphae were fluorescently stained: *C. albicans* seeded monolayers were incubated with rabbit anti-Candida antibody (1:1000 in 1 × PBS; OriGene, Rockville, MD, USA) for 2 h and subsequently with a goat anti-rabbit-Alexa Fluor 488 antibody (1:2000 in 1 × PBS). Invaded hyphae were masked by the epithelial monolayer and remained unstained. Images were recorded using fluorescent and bright field channels in a Zeiss AxioObserver Z1 inverted fluorescence microscope (Carl Zeiss, Oberkochen, Germany). Experiments were carried out in triplicate and at least 10 fields and 40 hyphae per field were counted in total, then counted again for invaded hyphae (unstained). Invaded hyphae were divided by total hyphae in each field to determine percent invasion.

For hyphal growth assays, *C. albicans* seeded monolayers were prepared as described above and incubated for 30 min before non-adherent *C. albicans* cells were removed and washed five times with 1 × PBS and fixed with 4% formaldehyde. *C. albicans* cells were incubated with rabbit anti-Candida antibody (1:1000 in 1 × PBS; OriGene, Rockville, MD, USA) for 2 h and subsequently with a goat anti-rabbit-Alexa Fluor 488 antibody (1:2000 in 1 × PBS). Images were recorded using fluorescent and bright field channels in a Zeiss AxioObserver Z1 inverted fluorescence microscope (Carl Zeiss, Oberkochen, Germany). Measurements were made of at least 100 budding hyphae per sample.

For adhesion assays, *C. albicans* seeded monolayers were prepared as described above and incubated for 1 h before non-adherent *C. albicans* cells were removed and washed 3 times with 1 × PBS and fixed with 4% formaldehyde. *C. albicans* seeded monolayers were incubated with rabbit anti-Candida antibody (1:1000 in 1 × PBS; OriGene, Rockville, MD, USA) for 2 h and subsequently with a goat anti-rabbit-Alexa Fluor 488 antibody (1:2000 in 1 × PBS). Images were recorded using fluorescent and bright field channels in a Zeiss AxioObserver Z1 inverted fluorescence microscope (Carl Zeiss, Oberkochen, Germany). Images of at least 15 fields per sample were taken. Total *C. albicans* cells were counted in each field and averaged. Values were expressed as percent of control (*Ca* only) cells.

All image analyses were performed in the ImageJ software. Statistical differences between conditions were calculated using ordinary one-way ANOVA with Dunnett’s multiple comparison test for hyphal length and invasion assays, and Sidak’s test for adhesion assays.

### 4.10. Statistical Analysis

All of the calculations and statistical analysis were performed using GraphPad Prism 8.4 (GraphPad Software, San Diego, CA, USA).

## Data Availability

The data summarized in this study are available on request from the corresponding author.
